# Supplementary material for: A cross-sectional analysis of meteorological factors and SARS-CoV-2 transmission in 409 cities across 26 countries
Source: Nat Commun. 2021 Oct 13;12:5968. doi: 10.1038/s41467-021-25914-8 (PMC8514574; doi:10.1038/s41467-021-25914-8)
Supplement: Supplementary file 1 — Supplementary Information [file 41467_2021_25914_MOESM1_ESM.pdf]

A cross-sectional analysis of meteorological factors and SARS-CoV-2 transmission in 409 cities across 26 countries

Supplementary materials

Supplementary Methods

Dependence of sample size on maximum Oxford Government Response Tracker

We chose 70 as the maximum value of OxCGRT Government Response Index allowed in included days as a compromise between limiting confounding by government interventions and including enough cities to enable estimation of the associations studied. This choice was informed by the preliminary evaluation (see Supplementary Figure 7). Supplementary Figure 7a shows the % of the 502 total cities for which data was available, according to the chosen criteria (window length between 10-20 days and there were at least 10 cases) for given maximum OxCGRT Government Response Index values (ranging from 60 to 100). Supplementary Figure 7b shows the dependence of the number of days included in windows for different cut-off values. Windows were also required to include at least 10 days and to begin only when 10 cases had occurred. For this purpose, each day's OxCGRT Government Response Index value was lagged ten days, to allow for the incubation period and reporting delays. As the OxCGRT Government Response Index cut-off was lowered, the number of cities included and of days included in windows diminished. The sharp rise in the number of cities included by increasing the maximum allowed OxCGRT Government Response Index from 60 to 70 with diminishing increases beyond that suggested 70 as a sensible compromise. During the analysis we checked the possible residual confounding role of the capped OxCGRT index by including the value at the end of the time window (lagged by 10 days) as covariate in our model. After observing its strong effect, we retained this variable for all further analyses.

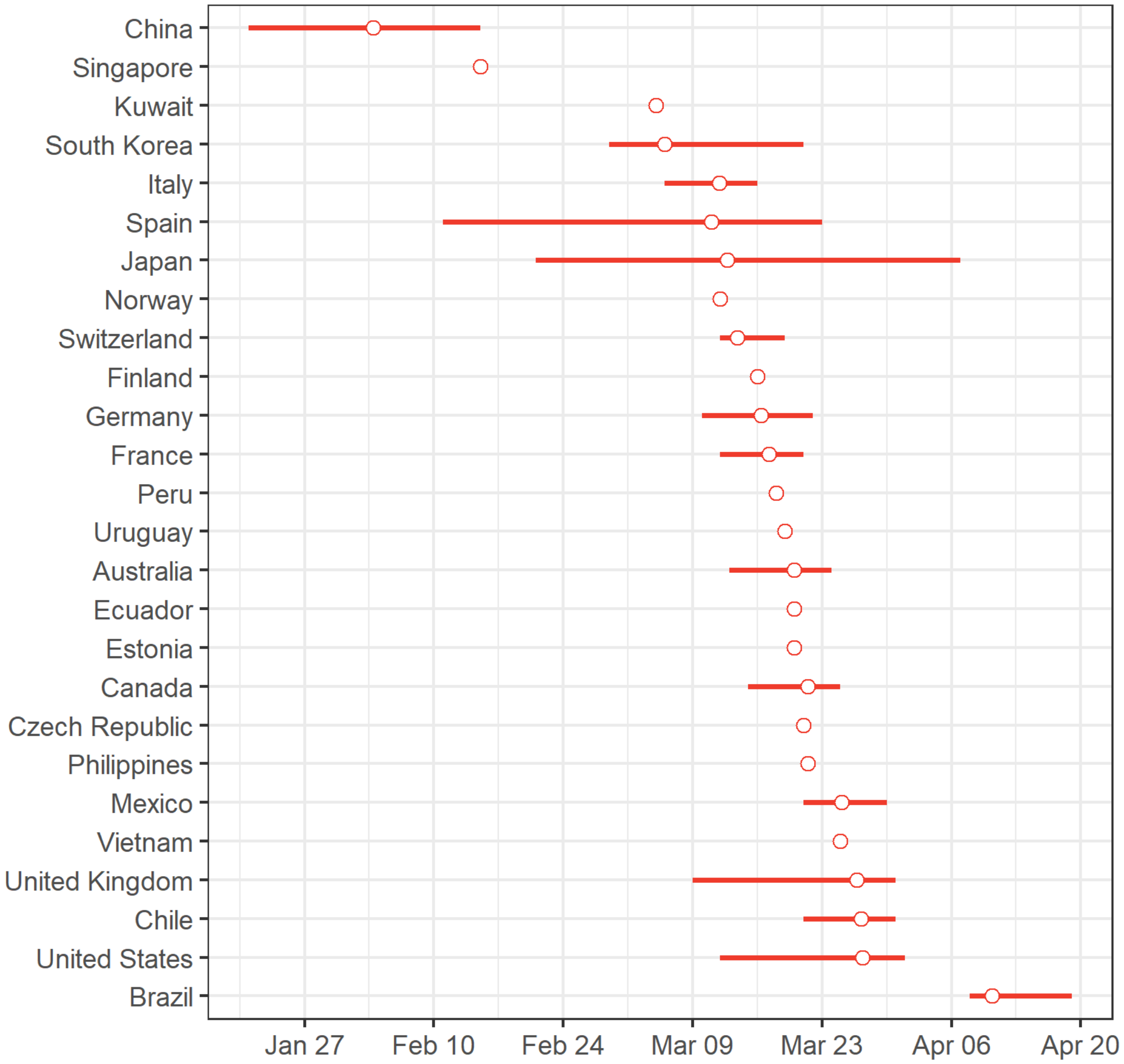

Supplementary Figure 1. Range (line) and mean (dot) observation day (midpoint of the time-window) for the cities within each of the 26 countries.

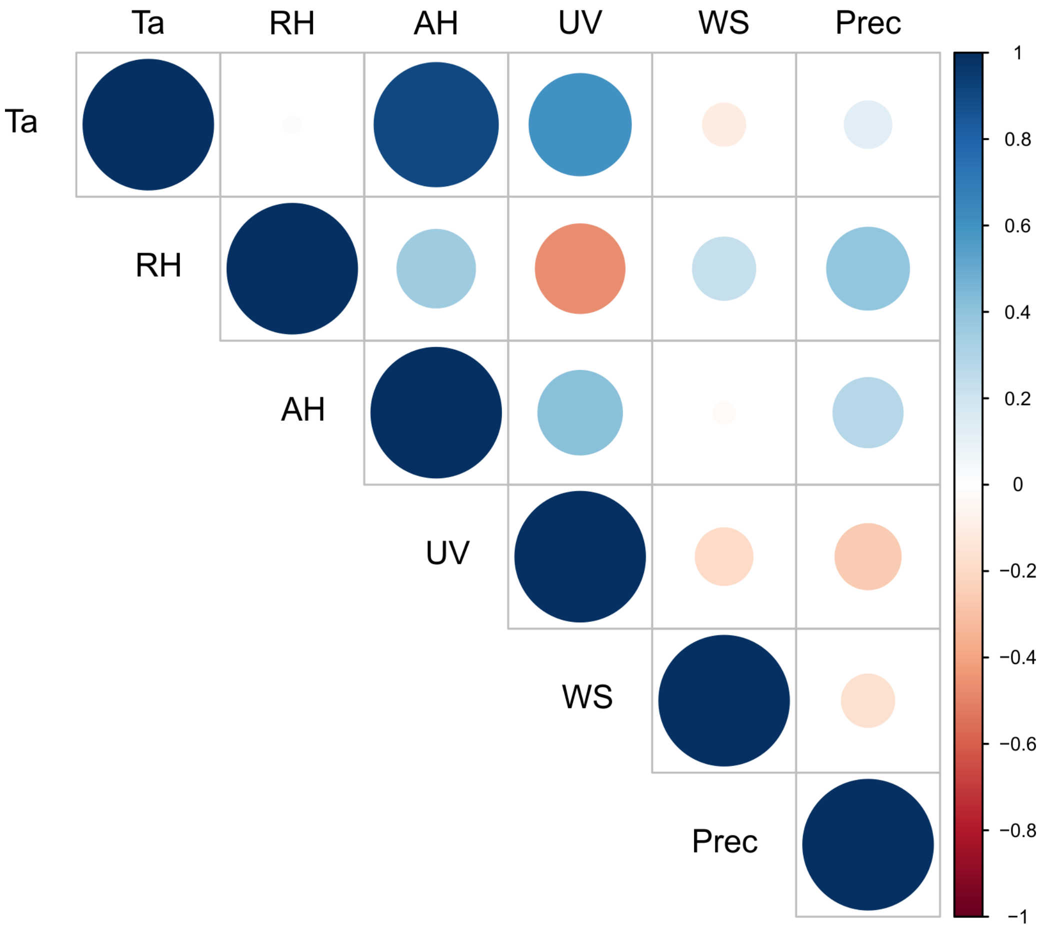

Supplementary Figure 2. Correlations between meteorological variables (Ta = Temperature, RH = Relative Humidity, AH=Absolute Humidity, UV=Surface solar radiation, WS=Wind speed, Prec=Total precipitations).

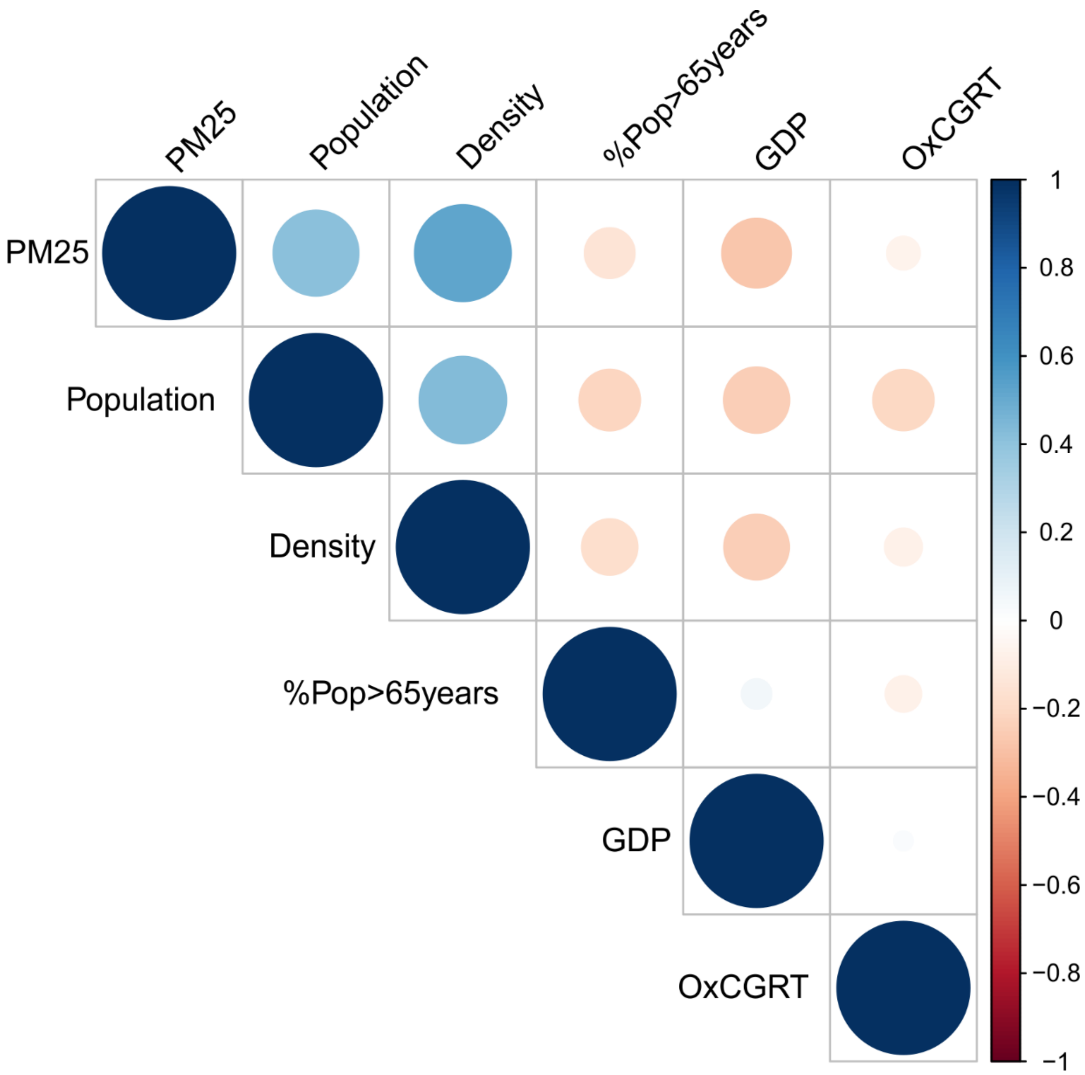

Supplementary Figure 3. Correlations a between city-level socio-demographic variables.

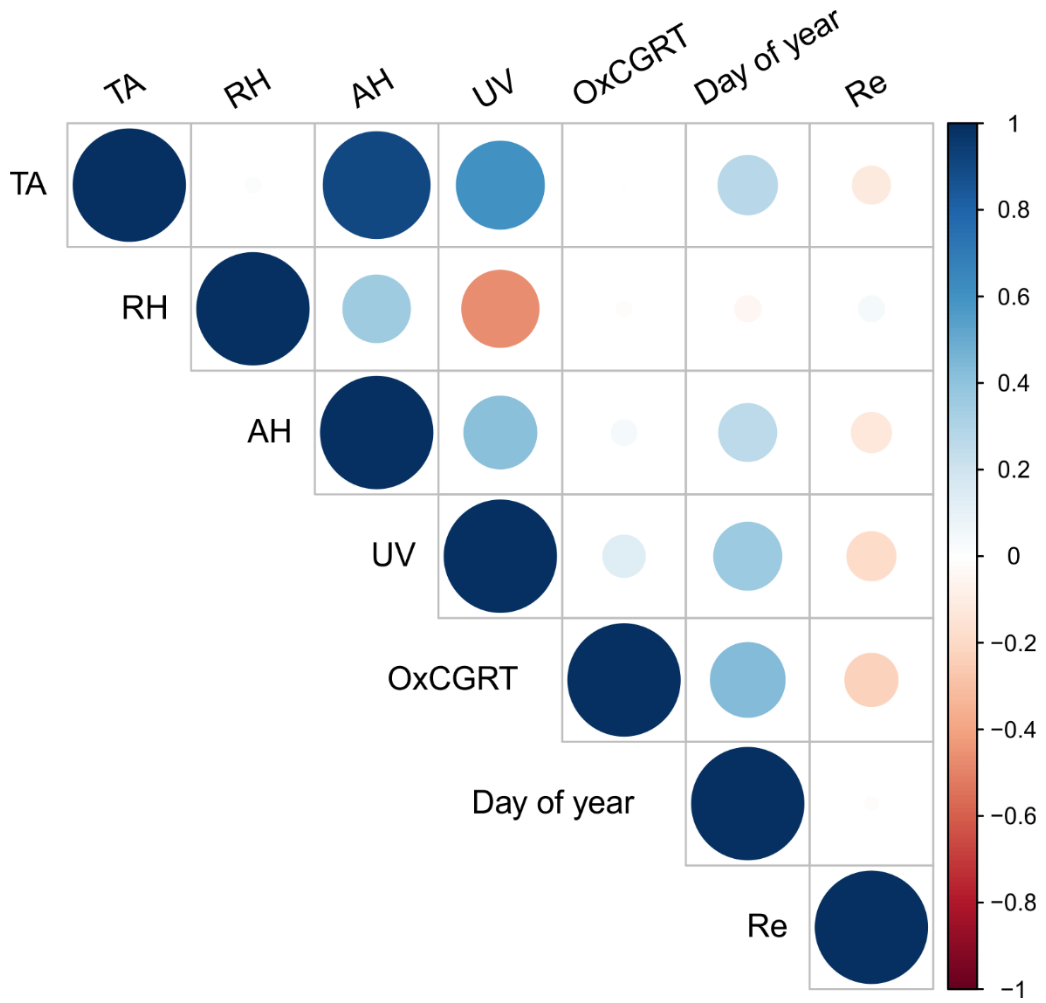

Supplementary Figure 4. Correlations between meteorological variables (Ta = Air temperature, RH = Relative humidity, AH=Absolute humidity, UV = Surface solar radiation), OxCGRT Government Response Index, day of the year, and Reproduction number (R_e).

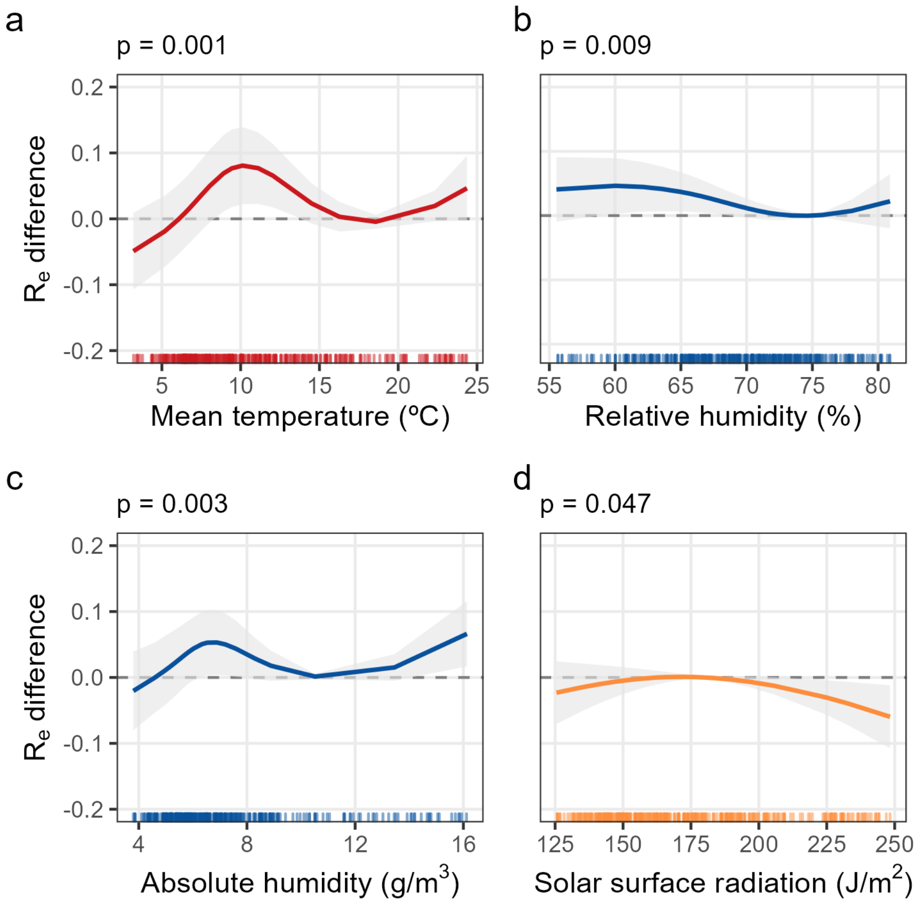

Supplementary Figure 5. Associations (with 95% confidence intervals) between (a) mean temperature (°C), (b) relative humidity (%), (c) absolute humidity (g/m3), and (d) Solar surface radiation with predicted Re difference when cities with $R_e < 1$ were excluded. Two-sided Wald test p-values and adjusted curves with 95% confidence intervals were obtained from multivariable meta-regression multilevel models adjusted by population (log scale), population density (log scale), GDP (log scale), % population >65 years of age, PM2.5 (μg/m^3, log scale) and OxCGRT Government Response Index, with cities nested within countries. The marginal distribution along the x axis represents the observed data for that covariate.

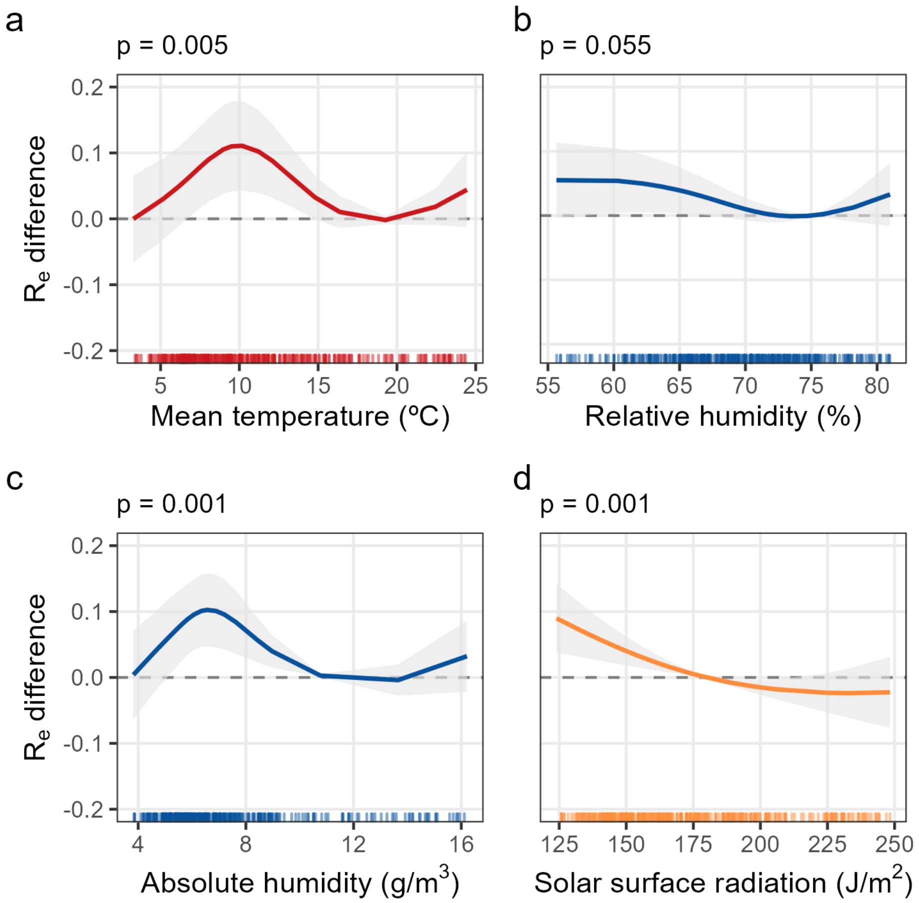

Supplementary Figure 6. Associations (with 95% confidence intervals) between (a) mean temperature (°C), (b) relative humidity (%), (c) absolute humidity (g/m3), and (d) Solar surface radiation with predicted Re difference when non-pharmaceutical interventions were not controlled for in the model. Two-sided Wald test p-values and adjusted curves with 95% confidence intervals were obtained from multivariable meta-regression multilevel models adjusted by population (log scale), population density (log scale), GDP (log scale), % population >65 years of age, PM2.5 ($\mu g/m^3$, log scale), with cities nested within countries. The marginal distribution along the x axis represents the observed data for that covariate.

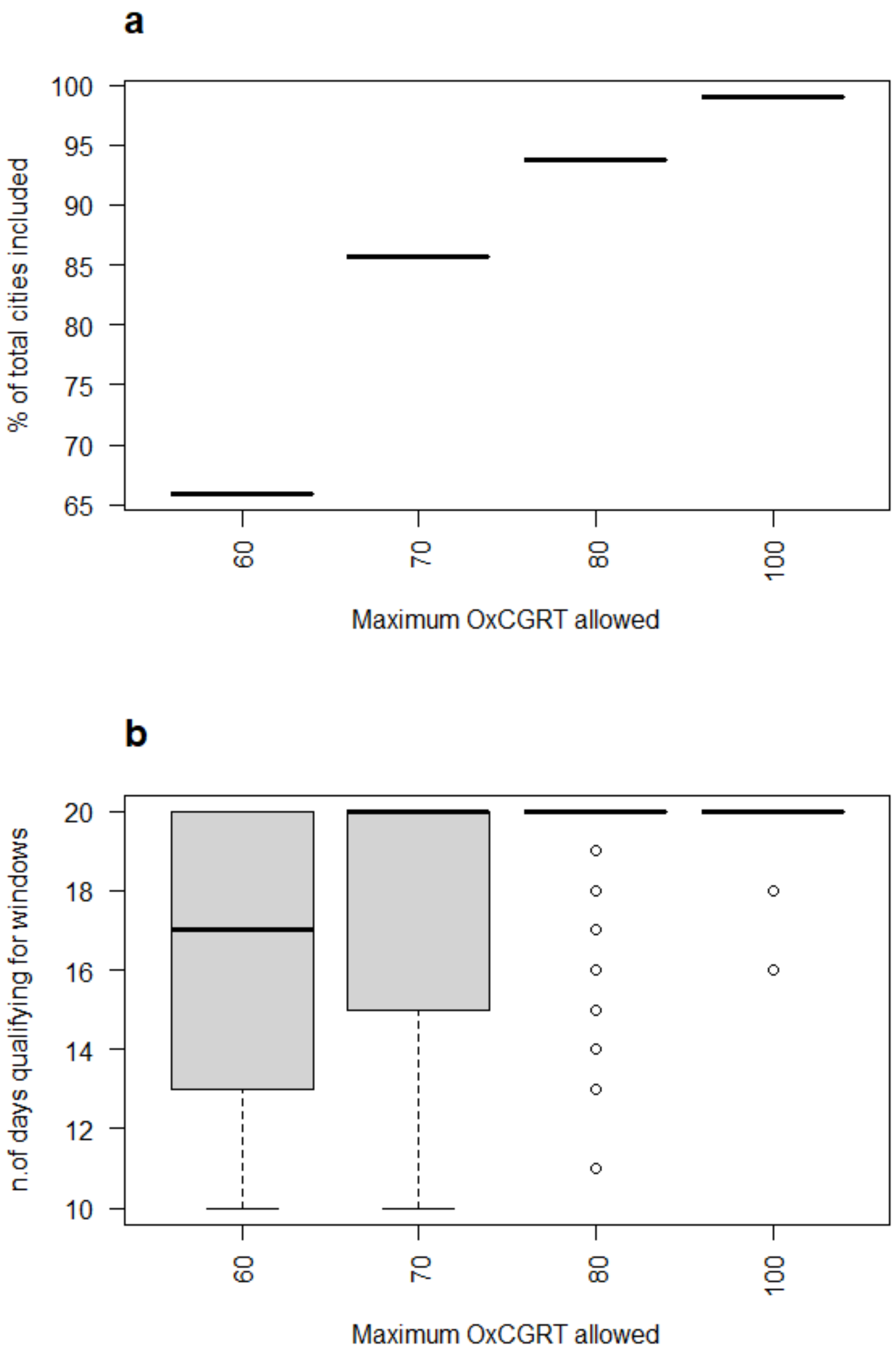

Supplementary Figure 7. (a) % of the 502 total cities for which data was available according to the chosen criteria (window length between 10-20 days and there were at least 10 cases) for given maximum OxCGRT Government Response Index values (ranging from 60 to 100). (b) number of days included in windows for a given cut-off value.

Supplementary Table 1. Source and COVID-19 case definition for the different countries.

**Country**	**Start date**	**End date**	**City definition**	**No. of cities**	**Source**	**Case Definition**
Australia	22/01/2020	04/06/2020	City	3	Health department website	Confirmed COVID-19 cases
Brazil	25/02/2020	04/06/2020	Municipality	18	https://covid.saude.gov.br/	The new confirmed COVID-19 numbers take into account the cases recorded from the previous day
Canada	25/01/2020	06/06/2020	Health Regions	17	https://github.com/ishaberry/Covid19Canada Berry I, Soucy J-PR, Tuite A, Fisman D. Open access epidemiologic data and an interactive dashboard to monitor the COVID-19 outbreak in Canada. CMAJ. 2020 Apr	The COVID-19 data includes confirmed and presumptive positive (i.e, probable) cases of COVID-19.
Chile	03/03/2020	12/06/2020	Regions	4	https://en.wikipedia.org/wiki/COVID-19_pandemic_in_Chile	Confirmed COVID-19 cases

China	22/01/2020	04/06/2020	City	17	nCov19 package in R	Confirmed COVID-19 cases
Czech Republic	29/02/2020	09/06/2020	Regions	1	The Ministry of Health of the Czech Republic - https://onemocneni-aktualne.mzcr.cz/api/v2/covid-19<br><br>Komenda M., Bulhart V., Karolyi M., et al. Complex reporting of coronavirus disease (COVID-19) epidemic in the Czech Republic: use of interactive web-based application in practice. *Journal of Medical Internet Research. 2020*, **22**(5), e19367.	RT-PCR confirmed cases per day
Ecuador	12/03/2020	15/05/2020	Provinces	2	Health authority	Confirmed COVID-19 cases
Estonia	26/03/2020	03/06/2020	County	1	Estonian Health Board - https://www.terviseamet.ee/et/koroonaviirus/avaandmed	Confirmed cases by clinical laboratory diagnostic tests.

Finland	01/03/2020	31/05/2020	Hospital districts	1	Finnish institute of health and welfare (THL)	All cases confirmed by laboratory testing. The date in the time-series refers to the date of taking the test
France	28/01/2020	08/06/2020	Departments	17	Santé publique France; data.gouv.fr	Until 19/3/2020 Confirmed cases. From 20/3/2020 Daily number of newly hospitalized persons

Germany	28/01/2020	31/05/2020	City	12	„Fallzahlen in Deutschland” of the Robert Koch-Institut (RKI) - Link to the dataset: https://www.arcgis.com/home/item.html?id=f10774f1c63e40168479a1feb6c7ca74	"Confirmed cases by clinical laboratory diagnostic tests. Infections confirmed by laboratory diagnostic evidence in case of a non-matching clinical picture (e.g. asymptomatic) are also included.
Italy	24/02/2020	04/06/2020	Provinces	23	Protezione civile	Confirmed COVID-19 cases
Japan	16/01/2020	31/05/2020	Prefectures	10	Health authority	Confirmed COVID-19 cases
Kuwait	22/01/2020	04/06/2020	Country	1	COVID-19 Dashboard by the Center for Systems Science and Engineering (CSSE) at Johns Hopkins University	RT-PCR positive nasopharyngeal swab

Mexico	01/01/2020	04/06/2020	States	8	https://datos.gob.mx/busca/dataset/informacion-referente-a-casos-covid-19-en-mexico	Confirmed COVID-19 cases
Norway	21/02/2020	26/05/2020	City	1	https://www.fhi.no/sv/smittsomme-sykdommer/corona/dags--og-ukerapporter/dags--og-ukerapporter-om-koronavirus/	Confirmed COVID-19 cases
Peru	06/03/2020	05/06/2020	Departments	18	Ministry of Health Peru (https://www.datosabiertos.gob.pe/group/datos-abiertos-de-covid-19)	Confirmed COVID-19 cases; test date
Philippines	09/03/2020	11/06/2020	City	4	https://doh.gov.ph/covid19tracker	RT-PCR confirmed cases per day
Romania	22/03/2020	31/05/2020	County	8	PRESS RELEASE, Strategic Communication Group, MINISTRY OF INTERNAL AFFAIRS	new cases of people infected with SARS – CoV – 2 (COVID – 19) these being cases that had not previously had a positive test

Singapore	23/01/2020	16/06/2020	City	1	Ministry of Health Singapore. (https://www.moh.gov.sg/covid-19/past-updates , https://www.moh.gov.sg/covid-19/situation-report)	Dates of confirmed COVID-19 cases
South Korea	20/01/2020	31/05/2020	Provinces	7	From http://ncov.mohw.go.kr/	People who diagnostic test positive for the virus, regardless of clinical manifestations.<br><br>All confirmed cases were registered in the KCDC Health and Disease Integrated Management System.

Spain	31/01/2020	21/06/2020	Provinces	52	https://cnecovid.isciii.es/covid19/#documentación-y-datos	Confirmed cases with clinical symptoms of acute respiratory infection of any severity with fever, cough or feeling of shortness of breath (other symptoms such as onychophagia, anosmia, ageustia, muscle pain, diarrhoea, chest pain or headache can also be considered) and with a positive result from a Diagnostic Test of Active Infection by SARS-CoV-2

Switzerland	01/01/2020	25/05/2020	Cantons	8	Federal Office of Public Health (FOPH, https://www.bag.admin.ch/bag/en/home.html) ; Federal Statistical Office (FSO, https://www.bfs.admin.ch/bfs/en/home.html)	Confirmed COVID-19 cases; date of testing
United Kingdom	30/01/2020	31/05/2020	LTLA	54	Public health England	The date in the time-series refers to the date the specimen was taken from the person being tested
United States	22/01/2020	04/06/2020	City	211	COVID-19 Dashboard by the Center for Systems Science and Engineering (CSSE) at Johns Hopkins University	Confirmed COVID-19 cases
Uruguay	24/02/2020	15/06/2020	Departments	1	Epidemiology Section of the Ministry of Health	Date start of symptoms
Vietnam	23/01/2020	19/06/2020	Provinces	2	Health authority	Confirmed COVID-19 cases

Supplementary Table 2. City-level socio-economic, demographic and pollution indicators.

**Indicator**	**Source**	**Year**
Total population (persons)	Worldcities database	2015
Population density (persons per km^2)	Worldcities database	2015
Population, % (population > 65 years)	OECD	2018
GDP per capita (US$)	OECD	2016;2018
$PM_{2.5}$ ($\mu g/m^3$)	CAMS Near real time	2020 (Covid window)

Supplementary Table 3. Mean, standard deviation (SD) and range (min and max) of the effective reproduction number, meteorological and city-level variables calculated in the 409 cities.

Variable	Mean	SD	min	max
Outcome				
R_e	1.43	0.19	0.70	2.11
Meteorological				
Mean temperature (°C)	11.27	6.66	-8.54	29.18
Relative humidity (%)	68.49	8.86	24.74	89.38
Absolute humidity (g/m^3)	7.65	3.97	1.88	22.19
Surface solar radiation downwards (J/m^2)	175.36	40.17	89.08	307.79
Wind speed (km/h)	2.88	1.15	0.63	7.30
Total precipitation (m/day)	2.46	2.23	0.00	21.07
City characteristics				
$PM_{2.5}$ (µg/m^3)	10.0	9.5	3.4	87.1
Total population (persons)	1,309,744.7	3,154,340.3	3,478.0	2,6174,599.0
Population density (persons per km^2)	4,157.4	5,397.8	42.0	53,108.1
Population, % pop >65 years	13.1	4.5	3.0	27.2
GDP per capita (US$)	37,752.2	14,922.3	3,168.0	101,375.0
Gross Value Added per capita (US$)	70,981.0	21,227.4	14,647.0	366,027.5

Non pharmaceutical Interventions				
Oxford Government Index	55.2	13.8	5.8	69.9

Supplementary Table 4. Sequence of multilevel meta-regression models.

**Model**	**Model terms**	**LogLik**	**likelihood ratio test R^2**	**I^2 (%)**	**Two-sided p-value**
Model A	Only random effects (city and country)	107.6483		66.2	
Model B	Model A + Oxford government index	149.0627	18.3	62.7	<0.0001
Model C	Model B + City-level covariates	153.8145	20.2	55.8	<0.0001
Model D1	Model C + Mean temperature (°C)	160.1787	22.7	53.3	0.014
Model D2	Model C + Relative humidity (%)	157.6298	21.7	55.3	0.058
Model D3	Model C + Absolute humidity (g/m^3)	159.0228	22.2	53.3	0.036
Model D4	Model C + Surface solar radiation downwards (J/m^2)	155.4314	20.8	55.7	0.208
Model D5	Model C + Wind speed (m/s)	155.7435	21.0	55.0	0.152
Model D6	Model C + Total precipitation (m/day)	154.7431	20.6	55.4	0.175
Model D7	Model D1 without Oxford government index	126.6178	8.9	58.6	
Model D8	Model D1 without City-level covariates	156.6395	21.3	59.3	

likelihood ratio test R^2 calculated as 1-exp(-2/409 x ($logLik_m$-$logLik_0$)), where logLikm is the log-likelihood of the model of interest and logLik0 is the log-likelihood from a null model including only city and country random effect (i.e. Model A).

Supplementary Table 5. Sensitivity analysis: p values for each experiment.

	Mean temperature (°C)	Absolute humidity (g/m^3)	Relative humidity (%)	Surface solar radiation downwards (J/m^2)	Wind speed (m/s)	Total precipitation (m)
Model presented in Table 2 (main text) (n = 409)	0.014	0.036	0.058	0.208	0.152	0.175
Cities with OxCGRT <60 (n = 129)	0.001	0.038	0.454	0.370	0.018	0.036
No Adjustment by OxCGRT (n = 409)	0.005	0.001	0.055	0.001	0.202	0.158
No lagged OxCGRT (n = 409)	0.016	0.035	0.107	0.260	0.132	0.256
Country as fixed effect (n = 409)	0.018	0.011	0.058	0.297	0.148	0.155
10 days lagged exposure variables (n = 409)	0.001	0.126	0.037	0.009	0.722	0.209
Models also adjusted by day of the year (n = 409)*	0.015	0.036	0.060	0.210	0.151	0.174
Only Cities with R>=1 (n = 399)	0.001	0.003	0.009	0.047	0.186	0.182
Excluding China and Brazil (n = 380)	0.011	0.156	0.049	0.332	0.189	0.880
Non tropical cities (n = 386)	0.019	0.201	0.063	0.199	0.185	0.699
Tropical cities (n = 23)	0.198	0.667	0.882	0.501	0.633	0.880
Northern hemisphere (n = 381)	0.036	0.355	0.054	0.294	0.192	0.774
Southern hemisphere (n = 28)	0.456	0.666	0.606	0.992	0.223	0.992
Only cities with latitude < 45 degrees (n = 308)	0.021	0.055	0.066	0.211	0.028	0.221

p values were obtained from multivariable meta-regression multilevel models adjusted by population (log scale), density (log scale) , GDP (log scale), % population > 65 years, $PM_{2.5}$ (log scale) and OxCGRT government response index with cities nested within countries.

*p values were obtained from multivariable meta-regression multilevel models adjusted by population (log scale), population density (log scale), GDP (log scale), % population > 65 years, $PM_{2.5}$ (log scale), OxCGRT oxford government response index and day of the year, with cities nested within countries.
