# Supplementary material for: A cross-sectional analysis of meteorological factors and SARS-CoV-2 transmission in 409 cities across 26 countries
Source: Nat Commun. 2021 Oct 13;12:5968. doi: 10.1038/s41467-021-25914-8 (PMC8514574; doi:10.1038/s41467-021-25914-8)
Supplement: Supplementary file 3 — Reporting Summary [file 41467_2021_25914_MOESM3_ESM.pdf]

nature research

Corresponding author(s): Rachel Lowe
Francesco Sera

Last updated by author(s): Aug 16, 2021

Reporting Summary

Nature Research wishes to improve the reproducibility of the work that we publish. This form provides structure for consistency and transparency in reporting. For further information on Nature Research policies, see our Editorial Policies and the Editorial Policy Checklist.

Statistics

For all statistical analyses, confirm that the following items are present in the figure legend, table legend, main text, or Methods section.

n/a	Confirmed	
☐	☒	The exact sample size (*n*) for each experimental group/condition, given as a discrete number and unit of measurement
☒	☐	A statement on whether measurements were taken from distinct samples or whether the same sample was measured repeatedly
☐	☒	The statistical test(s) used AND whether they are one- or two-sided<br>*Only common tests should be described solely by name; describe more complex techniques in the Methods section.*
☐	☒	A description of all covariates tested
☒	☐	A description of any assumptions or corrections, such as tests of normality and adjustment for multiple comparisons
☐	☒	A full description of the statistical parameters including central tendency (e.g. means) or other basic estimates (e.g. regression coefficient) AND variation (e.g. standard deviation) or associated estimates of uncertainty (e.g. confidence intervals)
☐	☒	For null hypothesis testing, the test statistic (e.g. F, t, r) with confidence intervals, effect sizes, degrees of freedom and P value noted<br>*Give P values as exact values whenever suitable.*
☒	☐	For Bayesian analysis, information on the choice of priors and Markov chain Monte Carlo settings
☐	☒	For hierarchical and complex designs, identification of the appropriate level for tests and full reporting of outcomes
☐	☒	Estimates of effect sizes (e.g. Cohen's *d*, Pearson's *r*), indicating how they were calculated

Our web collection on statistics for biologists contains articles on many of the points above.

Software and code

Policy information about availability of computer code

Data collection	COVID-19 data were downloaded from publicly available repositories or obtained from health agencies and data management was performed using Microsoft Excel 2019.<br>Meteorological variables (mean temperature, dew point temperature, solar radiation, wind components and precipitation) were derived from ERA5 reanalysis product "https://cds.climate.copernicus.eu/cdsapp#!/search?type=dataset". The data were downloaded in NetCDF format and processed with R version 4.0.3<br>Pollution levels (PM2.5) was derived from CAMS near real time "https://apps.ecmwf.int/datasets/data/cams-nrealtime/levtype=sfc/". The data were downloaded in NetCDF format and processed with R version 4.0.3.<br>the OxCGRT Government Response Index was downloaded from the public repository "https://github.com/OxCGRT/covid-policy-tracker/raw/master/data/OxCGRT_latest.csv" (downloaded Aug 3 2020), and data management was performed using R version 4.0.3.<br>Socio-economic and demographic characteristics were extracted from the OECD Regional and Metropolitan database "http://www.oecd.org/regional/regional-policy/regionalstatisticsandindicators.htm" and Worldcities database and data management and was performed using Microsoft Excel 2019.<br>Quality checks, data harmonization and linkages among the different dataset were performed using R version 4.0.3.
Data analysis	All statistical analysis were performed using R version 4.0.3. Relative humidity (RH; %) was calculated using the R “humidity” 0.1.5 package. We used the package EpiNow2 1.3.2 to calculate the effective reproduction number Re, and the package mixmeta 1.1.0 for fitting multilevel meta-regression models.<br>The custom code developed in the study to perform the city-level main analysis is available in the following GitHub repository: https://github.com/fsera/COVIDWeather

For manuscripts utilizing custom algorithms or software that are central to the research but not yet described in published literature, software must be made available to editors and reviewers. We strongly encourage code deposition in a community repository (e.g. GitHub). See the Nature Research guidelines for submitting code & software for further information.

Data

Policy information about availability of data

All manuscripts must include a data availability statement. This statement should provide the following information, where applicable:

- Accession codes, unique identifiers, or web links for publicly available datasets
- A list of figures that have associated raw data
- A description of any restrictions on data availability

COVID-19 data were downloaded from publicly available repositories or obtained from health agencies. COVID-19 data for Australia, Brazil, Canada, Chile, China, Czech Republic, Estonia, Finland, Germany, Italy, Kuwait, Mexico, Norway, Peru, Philippines, Romania, South Korea, Spain, United Kingdom, United States and Vietnam are publicly available. COVID-19 data for Japan and Singapore are available upon request. COVID-19 Data for France, Switzerland and Uruguay were obtained by a specific request to health agencies and are not publicly available.

Meteorological variables (mean temperature, dew point temperature, solar radiation, wind components and precipitation) were derived from ERA5 reanalysis product "https://cds.climate.copernicus.eu/cdsapp#!/search?type=dataset".

Pollution levels (PM2.5) was derived from CAMS near real time "https://apps.ecmwf.int/datasets/data/cams-nrealtime/levtype=sfc/".

the OxCGRT Government Response Index was downloaded from the public repository "https://github.com/OxCGRT/covid-policy-tracker/raw/master/data/OxCGRT_latest.csv" {downloaded Aug 3 2020).

Socio-economic and demographic characteristics were extracted from the OECD Regional and Metropolitan database "http://www.oecd.org/regional/regional-policy/regionalstatisticsandindicators.htm" and Worldcities database.

Data were processed and harmonized at city-level. The city-level data used in the main and supplementary analysis of the paper are available in the GitHub directory
https://github.com/fsera/COVIDWeather/

Field-specific reporting

Please select the one below that is the best fit for your research. If you are not sure, read the appropriate sections before making your selection.

☐ Life sciences ☐ Behavioural & social sciences ☒ Ecological, evolutionary & environmental sciences

For a reference copy of the document with all sections, see nature.com/documents/nr-reporting-summary-flat.pdf

Ecological, evolutionary & environmental sciences study design

All studies must disclose on these points even when the disclosure is negative.

Study description

We used a two-stage ecological approach to examine the impact of meteorological variables on SARS-CoV-2 transmission. in the first stage we estimated the effective reproduction number (Re) early in the epidemic in 409 locations (city or small region) within 26 countries. In the second stage, we estimated the association between city- level Re (allowing for standard errors) with meteorological variables (mean temperature, relative and absolute humidity, solar radiation, wind speed and precipitation), controlling for confounding by total population, population density, GDP per capita, percentage of population >65 years, PM2.5, and non-pharmaceutical interventions (OxCGRT Government Response Index). The analysis was performed considering the two-level (cities and countries) structure of the data using a multilevel meta-regression model

Research sample

Data in this study were obtained from the well-established MCC Collaborative Research Network. The current MCC network covers 750 cities in 43 countries/regions. For this study, 26 countries provided daily time-series of COVID-19 cases for a total of 502 locations (cities or small regions). COVID-19 data were downloaded from public available repository or obtained from health agencies. The MCC network provided access to COVID-19 data at the city (or small region) level. Analysis case data at this fine spatial scale reduces information bias and confounding compared to using large regions or country level data.

Sampling strategy

The MCC network provided access to COVID-19 data at the city (or small region) level. Despite the opportunistic nature of the sample, we achieved a reasonable global coverage. Overall, we collected 2,771,137 COVID-19 cases, representing 44.8% of the cumulative cases registered by 31 May 2020 in the Johns Hopkins database "https://coronavirus.jhu.edu/map.html".

Data collection

The research data are
1) Time-series of COVID-19 confirmed cases collected in 502 cities within 26 countries. Whenever possible COVID-19 data were downloaded from existing public repository. For some countries data were obtained from health authorities. The downloaded data were processed and harmonized by FS.
2) time-series of meteorological variables (mean temperature, relative and absolute humidity, solar radiation, wind speed and precipitation) derived from ERA5 reanalysis product. The data were downloaded by RS in NetCDF format and processed with R version 4.0.3.
3) Time series of Pollution levels (PM2.5). The data were derived from CAMS near real time. The data were downloaded by FS in NetCDF format and processed with R version 4.0.3.
4) Time fixed city level covariates derived from the OECD regional and metropolitan and World Cities database. The data were downloaded and processed by FS.

	5) the OxCGRT Government Response Index was downloaded from the public repository and data management was performed by BA using R version 4.0.3.
Timing and spatial scale	time series data were available from 1st January to 31st May 2020. The data were collected for 502 cities (or small regions) in 26 countries.
Data exclusions	We collected time-series COVID-19 data for 502 cities in 26 countries.<br>To limit potential confounding by interventions and temporal variation in case ascertainment, we selected a 20 day window early in the epidemic, starting after at least 10 cases had occurred in a 10-day period to reduce noise introduced by imported cases. We excluded days on which the Oxford COVID-19 Government Response Tracker (OxCGRT) Government Response Index exceeded 70, accepting reduced windows down to 10 days in length. Applying these conditions/restrictions reduced our dataset to 409 cities or small regions in 26 countries
Reproducibility	We perform several sensitivity analysis to evaluate the robustness of the results respect to analytic choices and sample selection.<br>In particular we repeated the analysis<br>1) excluding countries with higher non-pharmaceutical interventions<br>2) without adjusting by non-pharmaceutical interventions<br>3) considering country as fixed instead of random effect<br>4) excluding China and Brazil<br>5) in tropical and non-tropical locations<br>6) in Northern and Southern hemisphere locations<br>The association between mean temperature and the effective reproduction number holds across all the sensitivity analyses.
Randomization	This was an observational not randomized study. We consider the following city-level confounders total population, population density, GDP per capita, percentage of population >65 years, PM2.5, and non-pharmaceutical interventions (OxCGRT Government Response Index).
Blinding	Blinding was not necessary during data collection as we downloaded data already collected. During the analysis, model selection was guided by statistical goodness of fit criteria, including the Akaike information criterion (AIC).

Did the study involve field work? ☐ Yes ☒ No

Reporting for specific materials, systems and methods

We require information from authors about some types of materials, experimental systems and methods used in many studies. Here, indicate whether each material, system or method listed is relevant to your study. If you are not sure if a list item applies to your research, read the appropriate section before selecting a response.

Materials & experimental systems

n/a	Involved in the study	
☒	☐	Antibodies
☒	☐	Eukaryotic cell lines
☒	☐	Palaeontology and archaeology
☒	☐	Animals and other organisms
☒	☐	Human research participants
☒	☐	Clinical data
☒	☐	Dual use research of concern

Methods

n/a	Involved in the study	
☒	☐	ChIP-seq
☒	☐	Flow cytometry
☒	☐	MRI-based neuroimaging
